# Geographic Distribution and Incidence of Melioidosis, Panama^1^

**DOI:** 10.3201/eid2601.180870

**Published:** 2020-01

**Authors:** Ana B. Araúz, Katiana Castillo, Erika Santiago, Yarineth Quintero, Enrique Adames, Boris Castillo, Amalia Rodríguez-French, German Henostroza

**Affiliations:** Hospital Santo Tomás, Panama City, Panama (A.B. Araúz, K. Castillo, E. Santiago, Y. Quintero, E. Adames, A. Rodríguez-French);; Universidad de Panamá, Panama City (A.B. Araúz, E. Adames, A. Rodríguez-French);; Complejo Hospitalario Metropolitano Dr. Arnulfo Arias Madrid, Panama City (B. Castillo);; University of Alabama at Birmingham, Birmingham, Alabama, USA (G. Henostroza)

**Keywords:** melioidosis, *Burkholderia pseudomallei*, bacteria, Americas, Panama

## Abstract

Melioidosis is an infection caused by *Burkholderia pseudomallei*. Most cases occur in Southeast Asia and northern Australia; <100 cases have been reported in the Americas. We conducted a retrospective study and identified 12 melioidosis cases in Panama during 2007–2017, suggesting possible endemicity and increased need for surveillance.

*Burkholderia pseudomallei*, a gram-negative bacillus found in the environment of some tropical and subtropical regions, is the etiologic agent of melioidosis (*1**–**3*). Most melioidosis cases in the world are reported from Southeast Asia and northern Australia; only sporadic cases are reported from other regions (*4**–**6*). In the Americas, <100 acquired cases were identified from 1947 through June 2015. Only 3 cases were reported from Panama, 1 each in 1947, 1948, and 2011. However, cases were reported in Antioquia, Colombia (*1*,*7*,*8*), near the border with Panama. Melioidosis might be misdiagnosed and underreported because of the lack of diagnostic resources in the rural areas where cases are most likely to occur (*9*,*10*).

People become infected with *B. pseudomallei* through inoculation in compromised derma, inhalation, or ingestion. Some evidence suggests ingestion is associated with bacteremia, even though ingestion is considered an uncommon pathway (*2*,*3*,*11*). Clinical manifestations of melioidosis are diverse and may include localized cutaneous infection, pneumonia, involvement of bones and joints, intraabdominal abscesses, sepsis, and even death (*2*,*12*). Diagnosis is usually made through blood cultures, but the bacterium often is misidentified as *B. thailandensis* or *B. cepacia* (*10*,*13*). 

Current treatment for melioidosis includes an induction phase of 2–6 weeks with intravenous ceftazidime (or carbapenem for more severe cases), followed by a 2–6-month eradication phase using oral trimethoprim/sulfamethoxazole (TMP/SMX) or doxycycline. Doxycycline previously has been used for eradication, but recent studies suggest TMP/SMX is more effective (*2*,*3*). 

During the previous 10 years, cases of melioidosis have been identified in different regions of Panama. The aim of this study is to describe the clinical signs and symptoms and geographic distribution of melioidosis in Panama to elucidate the current status of the disease in the Americas.

## The Study

We conducted a retrospective review of medical records from 2007–2017 from 2 national tertiary level hospitals in Panama City. Hospital Santo Tomás and Complejo Hospitalario Metropolitano Dr. Arnulfo Arias Madrid (CHMDrAAM) are the 2 main referral hospitals for Panama and are in the capital city.

We reviewed specimen registries from the microbiology laboratories at each institution; we also identified 1 case from a poster presented at a national scientific meeting. We included patients who had a culture-positive report for *B. pseudomallei* and a clinical diagnosis of melioidosis at discharge. We excluded 2 patients with culture-positive results for *B. pseudomallei* because their clinical diagnoses were not related to their test results.

The microbiology laboratories of Hospital Santo Tomás and CHDrAAM identified *B. pseudomallei* strains from blood culture by using BacT/ALERT 3D Microbial Identification System (bioMérieux, https://www.biomerieux.com). Both laboratories also obtained isolates of *B. pseudomallei* from clinical specimens inoculated in Columbia agar prepared with 5% sheep blood and in MacConkey agar. Both laboratories used the VITEK 2 (bioMérieux) system to identify strains, which were then sent to the national reference laboratory at Instituto Conmemorativo Gorgas de Estudios de la Salud in Panama City, Panama, for microbiology confirmation and antimicrobial susceptibility testing.

We used a standardized form to collect data and then entered data into an Excel (Microsoft, https://www.microsoft.com) database for descriptive analysis. The Institutional Review Board of Hospital Santo Tomás reviewed and approved this study.

We identified 12 cases that occurred during 2007–2017: 8 in Hospital Santo Tomás and 4 in CHMDrAAM. We obtained medical records for all but 1 case, for which we obtained data from a poster presented at the 37th American College of Physicians Annual Central America Chapter Meeting in Panama City, Panama, in 2015 (*14*).

The mean age of cases was 50.3 years (SD ±12 years); most (9/12) patients were male. We noted bacteremia and sepsis in most (8/12) cases, pneumonia in 6 cases, and intraabdominal abscesses in 4 cases. Other signs and symptoms included endocarditis, meningitis, osteomyelitis, and septic arthritis (Table). Diabetes mellitus was the predominant risk factor. Most patients came from rural areas or suburbs of Panama City (Figure), and none reported travel outside of Panama.

**Table:** Clinical and epidemiologic characteristics of patients with melioidosis, Panama, 2007–2017*

Characteristics	Patient no.											
	1	2	3	4	5†	6	7	8	9	10	11	12
Age, y/sex	29/M	72/F	31/M	42/F	47/M	47/M	54/M	60/M	61/M	59/M	42/F	60/M
Area of origin	Oeste Pmá	Darién	Pmá	Darién	Oeste Pmá	Darién	Oeste Pmá	Coclé	Darién	Pmá	Colón	Pmá
Date of illness onset	Oct 2007	Aug 2009	Oct 2009	Nov 2009	NA	May 2012	Jul 2014	Aug 2015	Dec 2015	Aug 2016	Oct 2016	Jun 2017
Occupation	NA	NA	NA	NA	NA	NA	NA	Farmer	Farmer	Driver	House-wife	Retired
Risk factors	DM	DM	DM	DM	DM	CKD	DM	DM	DM	DM	DM	None
Duration of symptoms, wks	1	4	4	2	4	NA	NA	1	2	1	3	4
Symptoms												
Fever	Y	Y	Y	Y	Y	Y	Y	Y	Y	Y	Y	Y
Cough	Y	N	N	N	N	N	Y	Y	Y	Y	Y	N
Dyspnea	Y	N	Y	N	N	N	Y	Y	Y	Y	Y	N
Abdominal pain	N	Y	Y	Y	Y	N	Y	N	N	Y	N	N
Jaundice	N	N	N	Y	N	N	N	N	N	N	N	N
Seizures	N	N	N	N	N	N	N	Y	N	N	N	N
Joint pain	N	N	N	N	N	N	N	N	Y	N	N	Y
Diagnosis												
Bacteremia	Y	Y	N	N	Y	N	Y	Y	Y	Y	Y	N
Septic shock	N	N	N	Y	Y	N	Y	Y	Y	Y	Y	N
Pneumonia	Y	N	N	N	N	N	Y	Y	Y	Y	Y	N
UTI	Y	N	N	N	N	N	N	N	N	Y	N	N
Spleen abscess	N	Y	N	Y	N	N	N	N	N	N	N	N
Pancreatic abscess	N	N	N	N	Y	N	N	N	N	N	N	N
Liver abscess	N	N	Y	Y	N	N	N	N	N	N	N	N
Endocarditis	N	N	N	N	Y	N	N	N	N	N	N	N
Osteomyelitis	N	N	N	N	N	Y	N	N	N	N	N	N
Septic arthritis	N	N	N	N	N	N	N	N	Y	N	N	Y
Meningitis	N	N	N	N	N	N	N	Y	N	N	N	N
Positive culture	Blood, urine	Blood	LA	LA	Blood	TA	Blood	Blood, sputum, CSF	Blood, joint fluid	Blood, sputum, urine	Blood	SCA
Treatment	IPM, CAZ, TMP/ SMX	MEM, TMP/ SMX	IPM, TMP/ SMX	IPM, FEP, TMP/ SMX	CAR	IPM	MEM	MEM, CAZ	MEM	MEM, CAZ	MEM, CAZ	MEM
Outcome	Rec	Alive	Alive	Alive	Alive	Alive	Died	Died	Died	Died	Died	Alive

*CAR, carbapenem; CAZ, ceftazidime; CKD, chronic kidney disease; CSF, cerebrospinal fluid; DM, diabetes mellitus; FEP, cefepime; IPM, imipenem; LA, liver abscess; MEM, meropemen; N, no; NA, not available; Pmá, Panamá; SCA, sternoclavicular abscess; TA, tibial abscess; TMP/SMX, trimethoprim/sulfametoxazol; UTI, urinary tract infection; Y, yes.  †Information adapted from abstract of poster presented by E. Brid at American College of Physicians Central America Chapter Scientific Meeting, Panama City, Panama, 2015 Feb 27 (*14*).

**Figure:**
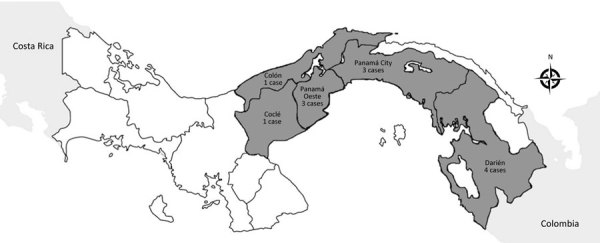
Regional distribution of melioidosis cases in Panama, 2007–2017.

All cases occurred during the rainy season, which is May–November in Panama. Five patients (41.7%) died while hospitalized; these patients had the most severe clinical manifestations of the disease, bacteremia, pneumonia, and septic shock, similar to cases reported from Central America (*15*). 

Rapid microbiologic identification of *B. pseudomallei* is necessary to initiate appropriate, life-saving treatments. However, laboratory results can take >48 hours, delaying appropriate antimicrobial drug therapy. Of the 7 patients in this study who survived, records showed they were treated with TMP/SMX or doxycycline, but the length of antimicrobial drug treatments were not noted in the records.

## Conclusions

The increase in reports of melioidosis in the Americas requires greater awareness of this disease among clinicians, especially those caring for patients with diabetes. Melioidosis often is misdiagnosed as pulmonary tuberculosis and scrofula (*10*); we found 2 misidentified clinical cases in our study. More studies are needed to identify specific high-risk areas and transmission routes in the Americas. Such insights can inform earlier clinical suspicion and guide the formulation of prevention strategies. 

Because the clinical signs and symptoms of melioidosis are nonspecific, microbiologic identification is crucial to diagnosis. Thus, improved laboratory capacity is critical to improve patient outcomes in affected areas to aid epidemiologic and antibiotic susceptibility surveillance efforts. Collaboration among countries in the region could drive efforts to describe the origins of this disease and the actual prevalence in the Americas.

Our study has limitations because we collected data retrospectively and only included the most severe cases in Panama. Melioidosis occurs more frequently in rural areas, and cases might not be identified because of the lack of laboratory or diagnostic tools. We provide a perspective on the processes that hinder our knowledge of this disease in Panama, such as lack of surveillance data and inadequate laboratory capacity. Our data justify the need for increased surveillance for melioidosis and reinforce the need for complete epidemiologic data and adequate strain storage for further genetic analysis. Epidemiologic studies of seroprevalence, environmental sampling, and increased access to PCR techniques and broth microdilution testing are needed to determine whether *B. pseudomallei* is endemic to Panama and to improve treatment outcomes.
